# Optimizing multi label student performance prediction with GNN-TINet: A contextual multidimensional deep learning framework

**DOI:** 10.1371/journal.pone.0314823

**Published:** 2025-01-22

**Authors:** Xiaoyi Zhang, Yakang Zhang, Angelina Lilac Chen, Manning Yu, Lihao Zhang

**Affiliations:** 1 College of Liberal Arts and Science, University of Illinois Urbana-Champaign, Urbana, IL, United States of America; 2 Industrial Engineering and Operations Research Department, Columbia University, New York, NY, United States of America; 3 Le Regent School, Crans-Montana, Switzerland; 4 Department of Statistics, Columbia University, New York, NY, United States of America; 5 Department of Information Engineering, The Chinese University of Hong Kong, Shatin, N.T., Hong Kong, China; Zhejiang Normal University, CHINA

## Abstract

As education increasingly relies on data-driven methodologies, accurately predicting student performance is essential for implementing timely and effective interventions. The California Student Performance Dataset offers a distinctive basis for analyzing complex elements that affect educational results, such as student demographics, academic behaviours, and emotional health. This study presents the GNN-Transformer-InceptionNet (GNN-TINet) model to overcome the constraints of prior models that fail to effectively capture intricate interactions in multi-label contexts, where students may display numerous performance categories concurrently. The GNN-TINet utilizes InceptionNet, transformer architectures, and graph neural networks (GNN) to improve precision in multi-label student performance forecasting. Advanced preprocessing approaches, such as Contextual Frequency Encoding (CFI) and Contextual Adaptive Imputation (CAI), were used on a dataset of 97,000 occurrences. The model achieved exceptional outcomes, exceeding current standards with a Predictive Consistency Score (PCS) of 0.92 and an accuracy of 98.5%. Exploratory data analysis revealed significant relationships between GPA, homework completion, and parental involvement, emphasizing the complex nature of academic achievement. The results illustrate the GNN-TINet’s potential to identify at-risk pupils, providing a robust resource for educators and policymakers to improve learning outcomes. This study enhances educational data mining by enabling focused interventions that promote educational equality, tackling significant challenges in the domain.

## Introduction

Online courses are becoming increasingly common due to the proliferation of digital platforms. This gives students more freedom and flexibility in their study. Measuring students’ progress and comprehension is challenging when teachers and students do not have regular one-on-one interactions. Lower test scores and reduced student involvement may be caused by a lack of touch, which can impair academic progress [1]. Therefore, gathering information from various digital sources and evaluating it is essential for resolving these issues. Using performance data, teachers may identify students at risk of falling behind or being left out, enabling them to intervene quickly and boost their performance [2].

Educational Data Mining (EDM) is crucial for identifying patterns in large educational datasets, analyzing student success factors, and boosting learning outcomes [3]. Assessing student attendance and engagement on MOOC platforms using data mining technologies shows a positive correlation between academic success and active participation [4]. Learning analytics is a specialized field that uses data mining to predict educational achievement by analyzing student behaviour [5]. A rising corpus of research emphasizes clickstream data, which tracks student activities in online learning settings, as a measure of academic achievement. Research suggests that neuro-fuzzy algorithms may accurately predict academic success using behavioural data [6, 7]. Most techniques concentrate on linear correlations, omitting contextual and behavioural data nuances. Nuanced student engagement and performance data are essential for complete academic assessments, requiring advanced modelling approaches [8, 9].

Current prediction methods cannot identify at-risk pupils early, limiting proactive measures. Many traditional methodologies only predict results after a course and restrict the use of interventions that might enhance student learning [10]. Previous research has used a binary framework to predict outcomes (e.g., pass or fail), which might ignore students’ different performance levels. Advanced learning possibilities may help excelling students, but binary systems generally overlook them beyond simple pass/fail criteria [11, 12]. This constraint highlights the need for models that account for the multi-label nature of student performance, which acknowledges that one kid may have various academic, behavioural, and social performance markers.

Several machine learning and deep learning algorithms, including decision trees, random forests, and logistic regression, are used in EDM to predict student demographics, academic scores, and engagement metrics [13]. Traditional models struggle with multidimensional, contextual, and sequential educational data, which are necessary for a comprehensive perspective of student performance. Deep learning models like Convolutional Neural Networks (CNNs) and Long Short-Term Memory (LSTM) networks have improved temporal patterns in educational data. Still, it faces issues representing complicated data type relationships, particularly in multi-label contexts. CNNs and LSTMs may struggle to manage the complex interplay between demographic and behavioural data [14].

This study presents GNN-Transformer-InceptionNet (GNN-TINet) to predict multi-label student performance. GNN-TINet uses GNN, Transformer topologies, and InceptionNet to find complex contextual links and interdependencies in multivariate, time-series student data. This method improves forecast accuracy and supports multi-label student outcomes, revealing each kid’s needs. The GNN-TINet system handles educational datasets using contextual adaptive imputation and frequency encoding to preserve data quality and relevance. Models may adjust to missing values and categorical variables by reacting to contextual correlations in the data during preprocessing, boosting prediction robustness and generalizability. GNN-TINet may tailor educational interventions for at-risk students and strong achievers who may benefit from advanced challenges. This research’s multi-label prediction system advances educational data mining (EDM) by integrating cutting-edge deep learning architectures with innovative preprocessing. This technique improves EDM by offering educators accurate information to boost student progress via tailored learning and educational equity.

1. Developed the GNN-Transformer-InceptionNet (GNN-TINet) model to increase student performance prediction accuracy and multi-label classification by combining the GNN, Transformer, and InceptionNet architectures.
2. Pioneered new feature selection and data balance methods, such as CBCE for synthetic data production and HCFS for prioritized feature ranking.
3. To evaluate the dependability of the model and its effect on student outcomes, two new performance measures are created: the Learning Impact Factor (LIF) and the Predictive Consistency Score (PCS).
4. Contributed to the EDM field by providing a comprehensive, high-performing pipeline for early student performance prediction, supporting targeted interventions for improved educational results.

The related Work section includes essential research on student performance from other researchers in the field. The Conceptual Framework section describes the mechanics of our approach for student performance, which uses a deep ensemble. The Experimental Data and Results section thoroughly discusses the experimental data obtained. The Conclusions and Future Directions section ends with a review of the results and suggestions for further study.

## Related work

Student performance prediction research is prevalent in EDM. Improved prediction accuracy has been achieved using classic machine learning and sophisticated deep learning methods. Decision trees (DT) and random forests (RF) have been used in various studies to predict student outcomes based on demographic and academic factors. These approaches work in many situations with multi-label predictions and contextual multidimensional data [13, 14].

A researcher in [15] used a decision tree technique to examine how demographic factors, notably age, affect academic achievement. This research does not include behavioural data, essential for understanding performance dynamics. A study [16] predicted student performance using demographic data but not time series data. Another survey by [17] found that extracurricular activities improve academic performance. Although accurate, their random forest model did not fully use multidimensional data sources. Many logistic regression studies examined family factors, including wealth and size [18]. Demographic factors affected performance, but behavioural data complicated things.

Deep learning has expanded EDM research. Using a CNN network, the author in [19] extracted temporal characteristics from clickstream and evaluation score data. This methodology enhanced prediction accuracy but concentrated on sequential data without addressing performance prediction’s multi-label nature. Using time-behavioral data, [20] created a hybrid deep-learning model, GritNet, to detect high-risk kids. Their forecasts were encouraging, but the model did not account for contextual interactions between characteristics, which may significantly alter predictions. One study [21] used an LSTM neural network and attention mechanism to predict student performance. This approach increased accuracy by concentrating on vital information, but multi-class categorization, essential for thorough performance evaluations, was complex.

Another study [22] used a transformer to convert learning behaviour data into sequential feature vectors for student performance prediction. This novel strategy increased prediction granularity, but it was necessary to investigate how demographic data may improve model accuracy. Researchers [23] suggested a multi-head attention model and SVM to pick relevant behavioural variables, which improved temporal accuracy but not multidimensional data integration. The author in [24] advanced using multi-topological graph neural networks (MTGNN) to represent student interactions. This technique increased relational dynamics knowledge but lacked time-series characteristics and contextual information, limiting its predictive power. Researchers in [25] used a time-series neural network to capture distinctive learning patterns in clickstream and evaluation data. However, binary categorization limited student performance analysis.

Existing models frequently work to include multidimensional and contextual data. The author in [26] used a K-NN regression model with decision trees but restricted feature sets, limiting the possibility of adding more dynamic data sources. The study [27] employed standard approaches to predict early dropout, which frequently did not use all the data. The author in [5] predicted student performance using machine learning techniques, including DenseNet. Their method enhanced accuracy. However, the research did not examine merging DenseNet with other models for a more robust prediction framework.

The author [28] used a ResNet to predict students’ degree completion with high recall and accuracy. However, single-dimensional data hampered the model’s capture of complicated feature relationships. In contrast, the author [29] suggested a bidirectional LSTM-attention mechanism hybrid deep neural network. This model excelled in contextual feature extraction but struggled with multi-label classification. According to the existing literature, advanced models that combine contextual, multidimensional data for student performance prediction are needed. Many studies have concentrated on individual performance variables but neglected demographic, behavioural, and academic interdependencies. The GNN-TINet system synthesizes these varied data sources to fill these gaps and maximize multi-label student performance prediction, improving educational interventions and results. The literature summary is described in Table 1.

**Table 1:** Literature review on student performance prediction.

Ref	Technique Used	Objective Achieved	Limitations
[13, 14]	Decision Trees (DT), Random Forests (RF)	Predict student outcomes based on demographic and academic factors	Often struggle with multi-label predictions and contextual multidimensional data.
[15]	Decision Tree	Examined the impact of demographic factors (age) on academic achievement	Lacks consideration of behavioural data critical for understanding performance dynamics.
[16]	Demographic Data	Predicted student performance	Did not integrate time-series data, limiting predictive capabilities.
[17]	Random Forest	Analyzed the effect of extracurricular activities on academic performance	Did not fully exploit multidimensional data sources.
[18]	Logistic Regression	Examined family factors, such as income and size	Did not address complexities introduced by behavioral data.
[19]	CNN	Extracted temporal characteristics from clickstream and assessment score data	Focused on sequential data without addressing multi-label prediction.
[20]	Hybrid Deep Learning Model (GritNet)	Detected high-risk students using time-behavioral data	Did not account for contextual interactions between features.
[21]	LSTM with Attention Mechanism	Improved prediction accuracy by focusing on key information	Struggled with multi-class categorization for detailed assessments.
[22]	Transformer	Converted learning behaviour data into sequential feature vectors	Needed exploration of how demographic data could enhance model accuracy.
[23]	Multi-Head Attention Model, SVM	Selected relevant behavioral variables to improve predictions	Did not effectively integrate multidimensional data.
[24]	Multi-Topological Graph Neural Networks (MTGNN)	Improved understanding of relational dynamics among students	Lacked time-series features and contextual information, limiting predictive capabilities.
[25]	Time-Series Neural Network	Captured unique learning patterns from clickstream and assessment data	Focused on binary classification, limiting comprehensive analysis of performance levels.
[26]	K-NN Regression with Decision Trees	Predicted student performance	Relied on limited feature sets, missing opportunities to include dynamic data sources.
[27]	Standard Approaches	Focused on early dropout prediction	Often failed to leverage the full spectrum of available data.
[5]	DenseNet	Enhanced prediction accuracy for student performance	Did not explore merging DenseNet with other models for improved robustness.
[28]	ResNet	Predicted students’ degree completion with high accuracy	Single-dimensional data hampered the capture of complex feature relationships.
[29]	Bidirectional LSTM with Attention	Excelled in contextual feature extraction	Struggled with multi-label classification.

## Proposed methodology

**Fig 1:**
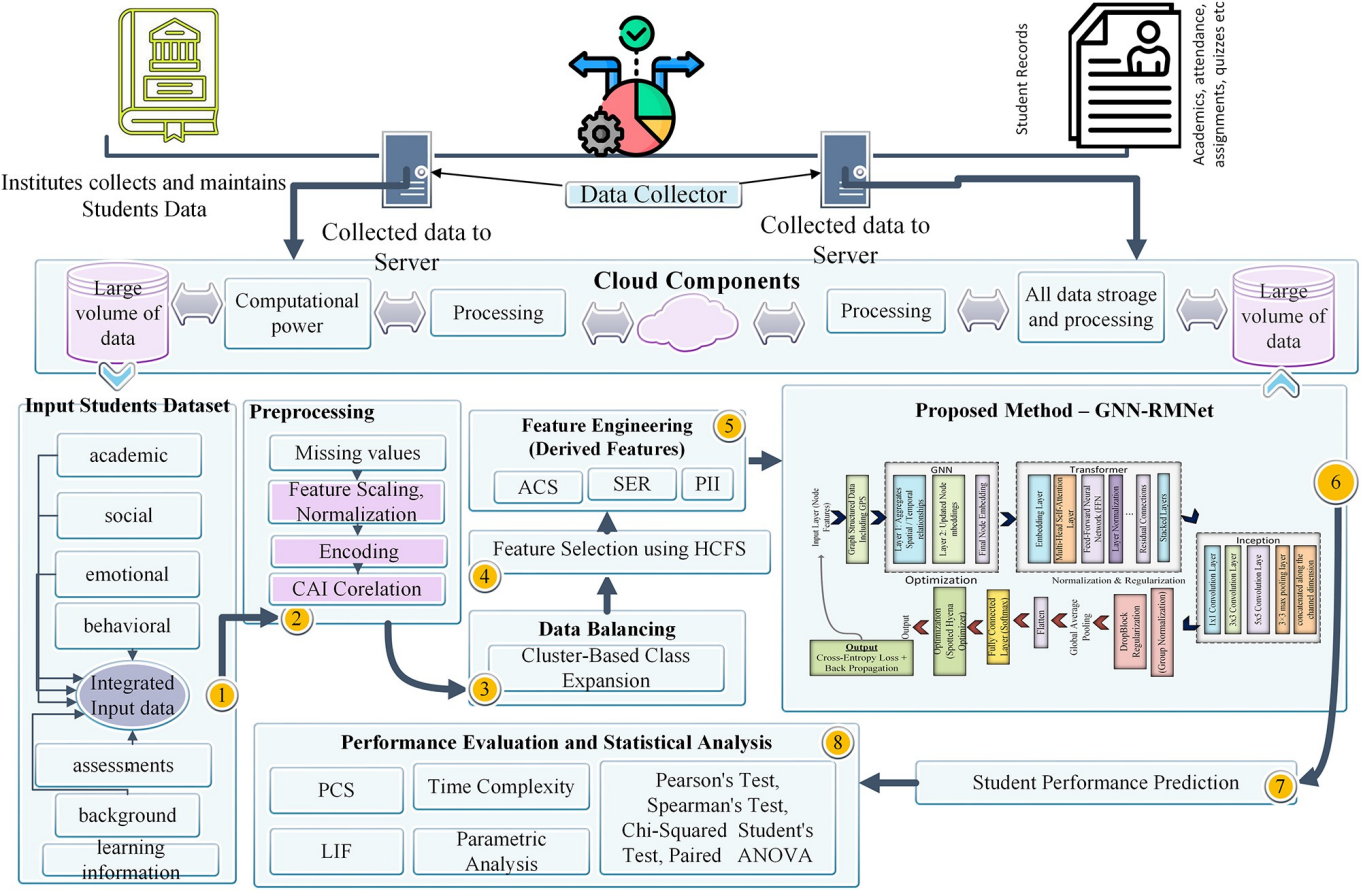
Proposed system framework.

The proposed method utilizes the GNN-Transformer-InceptionNet (GNN-TINet) model to improve multi-label student performance prediction by integrating sophisticated machine learning methodologies. The California Student Performance Dataset, a publicly accessible resource with many academic, behavioural, and emotional characteristics, is first preprocessed using advanced techniques like Contextual Adaptive Imputation and Contextual Frequency Encoding. The data is then organized into a graph representation, allowing the Graph Neural Network (GNN) to identify related patterns among students and their performance indicators. The GNN processes input via a message-passing approach to model global dependencies and then applies a Transformer network that uses self-attention methods. InceptionNet’s multi-scale architecture produces a complete data representation, which extracts many properties. Accuracy, predictive consistency score, learning impact factor, and many other metrics show that the model effectively identifies at-risk students and enables tailored educational interventions. Fig 1 shows the visual view of the proposed framework. The modules of the proposed framework are described in detail in subsequent sections.

### Dataset description

The dataset utilized in this study is the Student Performance Dataset (CSPD), which contains 97,000 data points from several California schools. It predicts student performance well using 36 characteristics and a broad range of student data. The publicly available data contains quantitative and qualitative data on students’ academic, social, emotional, and behavioural traits, which are included in this collection. All names, personal information, and sensitive data were anonymized or removed for privacy. [30] provides public access to the dataset. This website helps educators, data scientists, and policymakers improve educational outcomes by providing insights into numerous facets of student performance. CSPD was selected because it integrates technical and educational environment data, emotional and mental well-being indicators, social and behavioural data, real-time learning information, academic performance assessments, and student demographic and background facts. To examine student achievement comprehensively, the CSPD uses various data sources:

- Student Demographics and Background: Age, gender, socioeconomic status, parental education, and learning disabilities.
- Academic Data: Details on homework completion, project performance, GPA, and examination scores.
- Real-time learning data indicates class attendance, daily quiz outcomes, and study durations.
- Social and behavioural data include teacher feedback, motivation levels, and peer interactions.
- Mental health conditions, emotional awareness via wearables, and stress levels—data on emotional and psychological well-being.
- Technology and Learning Environment: Internet accessibility, use of instructional materials, and assessments of the educational environment.

This dataset was selected as it combines academic accomplishment, emotional well-being, social behaviour, and learning environments, which are crucial to understanding student outcomes. Academic, technological, and well-being data offer more exact and tailored student performance projections for intervention programs and instructional upgrades, enabling their implementation. Table 2 shows the dataset description and characteristics.

**Table 2:** Features of the CPSD.

SNo	Feature	Description
1	Student ID	Unique ID assigned to each student.
2	MentalHealth Status	Mental health status of the student (Good, Average, Poor).
3	Peer Influence Score	Influence of peers on the student’s performance, rated on a scale.
4	Age	Age of the student at the time of data collection.
5	Extracurricular Involvement	Participation in extracurricular activities like sports, clubs, etc.
6	Emotion Recognition Wearables	Emotional state tracked via wearables (Calm, Anxious, Stressed).
7	Average Subject Score	Average score across all subjects the student takes.
8	Teacher Feedback	Qualitative feedback teachers provide on the student’s progress.
9	Time Spent Studying	Average hours spent studying per week.
10	Subject Difficulty Rating	Self-reported or teacher-rated difficulty level of subjects.
11	Learning Disabilities	Whether the student has any diagnosed learning disabilities (Yes/No).
12	Learning Style	Preferred learning styles, such as Visual, Auditory, or Kinesthetic.
13	Quiz Test Scores	Scores obtained in quizzes or tests during the academic term.
14	School Environment Rating	Rating of the school’s learning environment on a scale.
15	Time Series Analysis	Tracking performance over time using time-series data.
16	Use of Educational Resources	Frequency of additional educational resources like libraries or online platforms.
17	Final Exam Scores	Final exam scores, typically representing end-of-term or board exams.
18	Device Usage Data	Time spent using digital devices for educational purposes.
19	Internet Access at Home	Availability at Internets/No).
20	Motivation Level	Self-reported level of motivation for academic activities.
21	Project Performance	Performance in group or individual projects, graded on a scale.
22	Participation in Class	Frequency of active participation in classroom discussions.
23	Peer Interaction	Frequency and quality of collaboration or peer interaction.
24	Homework Completion Rate	Percentage of homework assignments completed by the student.
25	GPA	Grade Point Average of the student, indicating overall academic performance.
26	Internal Exam Scores	Scores obtained in internal examinations.
27	Consistent Performance Patterns	Patterns of consistent performance across multiple exams.
28	Knowledge Gap Identification	Identification of specific knowledge gaps through AI-based analysis.
29	Socioeconomic Status	Student’s socioeconomic status, classified as Low, Middle, or High.
30	Highest Subject Score	Highest subject score achieved by the student.
31	Lowest Subject Score	Lowest subject score achieved by the student.
32	Parental Involvement	Frequency and extent of parental involvement in student’s education.
33	Parental Education Level	Highest education level attained by the parents.
34	Daily Quiz Scores	Scores from daily or weekly quizzes conducted during the academic term.
35	Subject Specific Improvement	Trends in improvement or degradation in specific subject performance.
36	Performance Category	Overall performance category (Very High, High, Medium, Low).

### Data preprocessing

Adequate dataset preparation is essential for early student performance prediction. This step handles missing values, encodes categorical variables, and normalizes numerical characteristics to prepare data for modelling [31]. A new preprocessing method emphasizes methodical and context-aware techniques. The new Contextual Adaptive Imputation approach addresses missing values. This approach improves feature relationship-based imputation by combining statistical methods with contextual data knowledge. We first check for disappeared entries, designated as *D*_*miss*_, which is the subset of *D* with missing values.

Start the CAI approach by finding features having a substantial link to the missing value feature. For missing values in feature *F*, we calculate a correlation score *C*(*F*, *G*) with all other features *G*:

$$\begin{array}{c} C\left(F,G\right)=\frac{\text{Cov}(F,G)}{\sigma_{F}\sigma_{G}} \end{array}$$ (1)

Cov signifies covariance, and *σ* represents feature standard deviation. For imputation, we keep characteristics with a correlation value over 0.3.

Weighted Mean Imputation is used for imputation. This technique weights features by association with the missing feature. For missing entries in feature *F*, the imputed value is determined as:

$$\begin{array}{c} F_{imputed}=\frac{\sum_{G}w\left(G\right)\cdot F_{G}}{\sum_{G}w\left(G\right)} \end{array}$$ (2)

*w*(*G*) is the correlation score-derived weight, and *F*_*G*_ represents the associated feature values for imputation. Each missing item is credited using the most appropriate attributes based on the data context.

The novel Contextual Frequency Encoding approach converts category information into numerical representations. We compute category frequency within relevant characteristics using this technique. For the categorical characteristic *C*:

$$\begin{array}{c} C_{encoded}=\frac{\text{count}(C_{i}|F_{j})}{\text{count}(F_{j})} \end{array}$$ (3)

The suitable feature is *F*_*j*_ and *C*_*i*_ represents a specific category. The model calculates category relevance by comparing category frequency to other characteristics.

For numerical normalization, Dynamic Range Scaling (DRS) is proposed. DRS scales are based on data distribution rather than min-max. DRS formula can be described as:

$$\begin{array}{c} F_{norm}=\frac{F-\mu }{\sigma } \end{array}$$ (4)

The local mean is denoted by *μ*, and the local standard deviation is denoted by *σ*; they are calculated using a sliding window of values around each occurrence. Feature robustness against outliers is enhanced by local context normalization. Afterwards, a novel method for detecting outliers is proposed: relative performance thresholding. This method determines d by using the average and standard deviation of the features. Extremely low or high numbers are considered outliers:

$$\begin{array}{c} \text{Outliers}=\{x:x>\mu +k\cdot \sigma \;\text{or}\;x<\mu -k\cdot \sigma \} \end{array}$$ (5)

By controlling or eliminating values according to feature distribution, *k* allows for personalized outlier detection when set as a user-defined sensitivity number, like two.

This preprocessing module uses novel techniques, including Relative Performance Thresholding, Dynamic Range Scaling, Frequency Encoding, and Contextual Adaptive Imputation. The dataset and early student, performance prediction model, are enhanced by accounting for missing values, normalizing numerical features, coding categorical variables, and overcoming outliers.

### Hierarchical contextual feature scoring (HCFS based feature selection)

HCFS selects features in a novel way that considers context, predictive power, and eliminating duplicate features. It selects data accordingly based on the independent forecasting of attributes and their relationships with other significant traits. Ratings might be improved by including hierarchical or grouped links of attributes. Determining the Mutual Information (MI) for each feature is the first step in the HCFS method. Feature data on Y is evaluated using MI. Here is the equation:

$$\begin{array}{c} MI\left(x_{j},y\right)=\int \int p\left(x_{j},y\right)\text{log}\frac{p(x_{j},y)}{p\left(x_{j}\right)p\left(y\right)}dx_{j}dy \end{array}$$ (6)

Although marginal probability distributions are *p*(*x*_*j*_), their combined distribution of probabilities for the feature *x*_*j*_ is *p*(*x*_*j*_). Higher MI-scored qualities have more associations, improving target relevance. HCFS considers contextual importance in the second review, unlike MI-based feature selection.

*p*(*x*_*j*_, *y*) represents the combined probability distribution of the feature *x*_*j*_ and the target *y*, whereas *p*(*x*_*j*_) and *p*(*y*) represent their marginal probability distributions. Higher MI score elements are preferred since they align more with the goal. Unlike previous MI-based feature selection methods, HCFS assesses contextual influence again.

$$\begin{array}{c} CIS\left(x_{j},C_{j}\right)=\frac{1}{|C_{j}|}\sum_{x_{k}\in C_{j}}MI\left(x_{j},x_{k}\right) \end{array}$$ (7)

In the context *C*_*j*_, the number of features is represented by—|C_j|—, and the mutual information between features *x*_*j*_ and *x*_*k*_ is quantified by *MI*(*x*_*j*_, *x*_*k*_). Because of this, HCFS may discover strong features and traits that benefit their group.

HCFS includes the Redundancy Penalisation (RP) phase to avoid selecting repetitive traits. Features that provide identical results are penalized to ensure that the final features contain diverse and non-redundant information. The redundancy penalty for feature *x*_*j*_ is calculated as follows:

$$\begin{array}{c} RP\left(x_{j}\right)=\sum_{x_{k}\in S}MI\left(x_{j},x_{k}\right) \end{array}$$ (8)

S is the previously selected collection of features, whereas *MI*(*x*_*j*_, *x*_*k*_) measures the mutual information between *x*_*j*_ and each *x*_*k*_ in the set. This redundancy reduction phase enhances the model’s performance by preventing overfitting caused by an excessive number of similar features.

The final stage of HCFS is integrating these calculations into an HCFS for each feature. Contextual interaction, redundancy penalty, and feature mutual information comprise the score:

$$\begin{array}{c} HCFS\left(x_{j}\right)=MI\left(x_{j},y\right)+\lambda_{1}CIS\left(x_{j},C_{j}\right)-\lambda_{2}RP\left(x_{j}\right) \end{array}$$ (9)

The relative importance of the contextual engagement score and the redundant penalty is decided by the weighting factors λ_1_ and λ_2_. The model employs the characteristics that rank highest according to their HCFS scores. HCFS emphasizes contextual interactions and hierarchical feature relationships, unlike conventional feature selection methods. It better captures the intricacy of multidimensional data since it groups attributes and analyzes their interconnections. Every step of the redundancy penalty process keeps the model efficient and prevents it from overfitting. Because it considers students’ academic, behavioural, emotional, and contextual aspects, educational data mining successfully employs HCFS in complicated datasets. HCFS enables one to put facts about ideas, emotions, and behaviours in perspective. It modulates for overlapping characteristics and ranks the top predictors to reduce repetition. The provided relevant and diverse feature sets improve model performance.

### Feature engineering

Feature engineering seeks to enhance a system’s predictive capabilities by combining or transforming current features and adding newly created ones. Three more features are added to the current ones to improve the ability of the Hierarchical Contextual Feature Scoring (HCFS) method to capture relationships between data points.

#### Academic Consistency Score (ACS)

ACS is derived from Grade Point Average (GPA), Internal Exam Scores, and Final Exam Scores. This function evaluates the reliability of a student’s results when examined via several techniques. The equation for it is:

$$\begin{array}{c} ACS=\frac{GPA+Internal\_Exam\_Scores+Final\_Exam\_Scores}{3} \end{array}$$ (10)

The ACS score provides academic stability By averaging student’s performance using important academic metrics,

#### Study Efficiency Ratio (SER)

The SER is determined by dividing the student’s homework completion rate by their study time. This tool compares the student’s study time to their assignment completion time to evaluate the effectiveness of their study habits.

$$\begin{array}{c} SER=\frac{Time\_Spent\_Studying}{Homework\_Completion\_Rate} \end{array}$$ (11)

A lower SER indicates that students complete homework with less studying time. On the other hand, an increased SER can suggest that the research methodologies used were inefficient.

#### Peer Influence Index (PII)

The PII consists of two scores—one for peer interaction and one for peer influence. This trait quantifies how much a student’s classmates influence their conduct and academic achievement.

$$\begin{array}{c} PII=Peer\_Interaction\times Peer\_Influence\_Score \end{array}$$ (12)

This derived characteristic lets one measure how much peer networks influence student achievement. Greater PII values point to more intense peer impact.

### Data balancing using Cluster-Based Class Expansion (CBCE)

The CBCE method generates synthetic instances in underrepresented classes obtained from tiny data point clusters, reducing class imbalance. This method guarantees that the newly generated synthetic data adheres to each class’s inherent feature distribution and variation while mitigating duplication in overrepresented classes by eliminating similar cases. The objective is to provide a balanced and diversified dataset to enhance model generalization for predicting student performance.

**Step 1: Clustering of data points within each class**. The first phase of CBCE entails grouping instances within each class according to their feature similarity. Let D denote the dataset, wherein the target variable comprises *m* classes: $C_{1},C_{2},\ldots ,C_{m}$. For each underrepresented class $C_{i}$, we implement a clustering technique (e.g., K-Means or DBSCAN) [32] to partition the instances into *k*_*i*_ clusters, referred to as $G_{1},G_{2},\ldots ,G_{k_{i}}$. Each cluster $G_{i}$ consists of feature vectors $z_{j}\in G_{i}$.

$$\begin{array}{c} G_{i}=\left\{z_{1},z_{2},\ldots ,z_{l}\right\},\;z_{j}\in C_{i} \end{array}$$ (13)

This clustering guarantees that data points are clustered according to natural patterns in the feature space, allowing for meaningful synthetic instance production.

**Step 2: Cluster boundary expansion for synthetic instance generation**. Generation of synthetic instances is the subsequent stage after the clusters within the minority class are identified. This is accomplished by expanding the boundaries of the clusters. The convex hull, which denotes the minimal convex boundary encompassing all cluster elements, is computed for each cluster $G_{i}$. The set of coordinates that define the cluster’s boundary is referred to as the convex hull:

$$\begin{array}{c} \text{ConvexHull}\left(G_{i}\right)=\left\{z_{1},z_{2},\ldots ,z_{p}\right\},\;z_{j}\in G_{i} \end{array}$$ (14)

To generate additional synthetic instances, we marginally extend the limits of the convex hull. Let $y\in \text{ConvexHull}(G_{i})$ denote a border point, and let $z_{G_{i}}$ represent a randomly selected point from inside the cluster. The synthetic instance *z*_new_ is produced using linear interpolation between the boundary point *y* and the interior point $z_{G_{i}}$:

$$\begin{array}{c} z_{\text{new}}=\beta z_{G_{i}}+\left(1-\beta \right)y,\;\beta \in \left[0,1\right] \end{array}$$ (15)

The interpolation factor *β* determines the extent of new instances beyond the current cluster bounds. We choose *β* depending on cluster variance to provide synthetic examples that match the natural distribution of data inside the class. Lower-variance clusters create examples closer to the centre, whereas higher-variance clusters yield more scattered synthetic points.

**Step 3: Conditional suppression of over-represented classes**. In over-represented classes, basic downsampling may result in significant data loss. CBCE employs a *conditional suppression* method to prevent this. For each instance *z*_*j*_ in the over-represented class $C_{i}$, we calculate its variance contribution *v*(*z*_*j*_), which quantifies the deviation of the instance from the mean of its cluster:

$$\begin{array}{c} v\left(z_{j}\right)=\left\|z_{j}-\mu_{G_{i}}\right\|,\;\mu_{G_{i}}=\frac{1}{|G_{i}|}\sum_{z_{j}\in G_{i}}z_{j} \end{array}$$ (16)

In this instance, $\mu_{G_{i}}$ represents the cluster mean, and *v*(*z*_*j*_) represents the contribution of each instance to the cluster’s spread. Remove instances with low variance contributions, which are closer to the cluster mean and redundant. Set *θ* as a suppression threshold to achieve desired diversity in the over-represented class:

$$\begin{array}{c} S\left(z_{j}\right)=\{\begin{array}{cc} 0 & \text{if}\;v(z_{j})<\theta \\ 1 & \text{if}\;v(z_{j})\geq \theta \end{array} \end{array}$$ (17)

Only instances with *S*(*z*_*j*_) = 1 are maintained, ensuring that the retained data points preserve the class’s variety while avoiding excessive repetition.

**Step 4: Iterative balance adjustment**. Class distributions are re-evaluated after each cycle of synthetic instance production and conditional suppression. Until the class ratios fall within a suitable range, clusters repeatedly grow and shrink. The chance of $V_{O}$ happening determines the proportion of class $C_{i}$ after each iteration; |F| is the total of all occurrences in the dataset.

$$\begin{array}{c} P\left(V_{o}\right)=\frac{|V_{o}|}{\left|F\right|} \end{array}$$ (18)

This cycle continues until all classes are represented, at which point it ends. The synthetic instances that comprise the final dataset that CBCE creates preserve various occurrences within the majority classes and successfully augment the data for the minority classes. For early student performance forecasting models to provide accurate and generalizable predictions, the dataset must be balanced while retaining each class’s inherent diversity and structure.

### Classification with GNN-Transformer-InceptionNet Network (GNN-TINet)

Graph Neural Networks (GNNs) [33, 34], Transformer Networks [35], and InceptionNet [36] are combined in the GNN-TINet network to categorize complex information, including student performance prediction efficiently. Transformer uses attention techniques to capture global relationships, InceptionNet enhances multi-scale feature extraction to diversify learned features, and GNN models the relational and graph-based data structure. The dataset is first shown in GNN-TINet as a graph G=(V,E), with *V* standing for nodes (such as students or devices) and *E* for edges (relationships between nodes). Each node $v_{j}\in V$ has a feature vector **f**_*j*_. A message-passing mechanism updates each node’s representation in the GNN. The message-passing update rule for node *v*_*j*_ at layer *l* + 1 is:

$$\begin{array}{c} {\text{f}}_{j}^{\left(l+1\right)}=\phi ({\text{W}}^{\left(l\right)}{\text{f}}_{j}^{\left(l\right)}+\sum_{k\in N(j)}{\text{W}}_{\rceil }^{\left(l\right)}{\text{f}}_{k}^{\left(l\right)}) \end{array}$$ (19)

${\text{f}}_{j}^{\left(l\right)}$ represents the feature vector of node *v*_*j*_ at layer *l*, N(j) represents its neighbors, and **W**^(*l*)^ and ${\text{W}}_{\rceil }^{\left(l\right)}$ are learnable weight matrices for nodes. This approach lets the GNN collect local and neighbourhood data and encode relational patterns.

After identifying minority class clusters, input GNN output into a Transformer network. Self-attention techniques help the Transformer represent global dependencies and interactions, capturing complicated dataset linkages. The self-attention mechanism is:

$$\begin{array}{c} \text{Att}\left(\text{W},\text{L},\text{B}\right)=\text{softmax}(\frac{{\text{WL}}^{\text{Y}}}{\sqrt{f_{j}}})\text{C} \end{array}$$ (20)

The key, query, and value matrices from the GNN output are denoted by **W**, **K**, **B** in this equation, whereas the essential vector dimensionality is represented by *f*_*j*_. This approach improves classification accuracy by guiding the model to focus on crucial interactions between nodes or between temporal sequences.

Inception processes Transformer output to boost the model’s capacity to learn varied characteristics. Each Inception block uses convolutional filters with varying kernel sizes (1x1, 3x3, 5x5) to capture characteristics at different scales. Calculating Inception module output:

$$\begin{array}{c} {\text{f}}_{\text{out}}=\text{Concat}\left({\text{f}}_{1\times 1},{\text{f}}_{3\times 3},{\text{f}}_{5\times 5},{\text{f}}_{\text{pool}}\right) \end{array}$$ (21)

**f**_1×1_, **f**_3×3_, **f**_5×5_ represent the outputs from convolutional layers with varying kernel dimensions, whereas **f**_pool_ is derived from a max-pooling layer. This multi-scale methodology allows the model to acquire features attuned to intricate and overarching patterns within the data.

The characteristics derived from the Inception module are flattened and then processed via fully linked layers for classification. The anticipated output $\hat{\text{y}}$ is expressed as:

$$\begin{array}{c} \hat{\text{y}}=\text{softmax}\left({\text{W}}_{\text{f}}{\text{f}}_{\text{out}}+{\text{b}}_{\text{f}}\right) \end{array}$$ (22)

**W**_f_ and **b**_f_ represent the weights and bias of the fully connected layer, assuring a probability distribution across classes. The GNN-TINet is trained to reduce cross-entropy loss:

$$\begin{array}{c} L=-\sum_{i=1}^{m}t_{i}\;\text{log}\left({\hat{y}}_{i}\right) \end{array}$$ (23)

Where *t*_*i*_ represents the actual label for instance *i* and ${\hat{y}}_{i}$ represents the expected probability. This complete design lets the GNN-TINet classify data using relational, temporal, and multi-scale aspects.

GNN-TINet is ideal for student performance prediction, where connections between students, instructors, and peers are critical, and network traffic analysis, where multi-scale feature extraction and relationship modelling are needed for anomaly identification. Integrating GNN, Transformer, and InceptionNet creates a strong and versatile architecture that can handle numerous categorization tasks.

### Performance evaluation metrics

The developed model for predicting early student performance is assessed using classic and innovative indicators customized to the educational setting [37–39]. Accuracy, precision, recall, and F1-score reveal the model’s student performance category classification performance. Accuracy is a simple measure of correctness, although class imbalance might underrepresent some performance areas. Precision and recall improve assessment by emphasizing expected categorization relevance. Precision measures how many optimistic predictions are correct, whereas recall measures the model’s ability to find all relevant occurrences. These measures are crucial in education because incorrectly labelling a difficult student might have far-reaching effects. The Learning Impact Factor (LIF) and the Predictive Consistency Score (PCS) are two new performance measures that complement existing ones. The PCS highlights the importance of consistent performance across evaluations by measuring the consistency of model predictions across time. Organized as follows:

$$\begin{array}{c} \text{PCS}=\frac{1}{N}\sum_{i=1}^{N}(\frac{1}{T_{i}}\sum_{t=1}^{T_{i}}I\left({\hat{y}}_{i,t}=y_{i,t}\right)) \end{array}$$ (24)

The equation includes N students, *T*_*i*_ predictions, anticipated performance (${\hat{y}}_{i,t}$), actual performance (*y*_*i*,*t*_), and the indicator function (I). If the indication is correct, it returns one; otherwise, it returns zero. Effective intervention techniques rely on the model’s ability to predict across assessments reliably; a better PCS demonstrates this. To determine how well the model foretells students’ long-term performance, especially in response to interventions or changes to their learning environment, the LIF uses a variety of metrics, as shown in the equation below.

$$\begin{array}{c} \text{LIF}=\frac{1}{N}\sum_{i=1}^{N}\frac{|\Delta y_{i}|}{|\Delta {\hat{y}}_{i}|+\epsilon } \end{array}$$ (25)

While the change in actual performance for student *i* is recorded by Δ*y*_*i*_ = *y*_*i*,*T*_ − *y*_*i*,0_, the expected performance change is shown by $\Delta {\hat{y}}_{i}=y_{i,T}-{\hat{y}}_{i,0}$. One avoids zero division using the tiny constant *ϵ*. The approach is valuable in classrooms if the LIF is sufficient, as it can identify significant variations in student performance.

In the performance assessment framework, the combination of PCC and LIF offers a comprehensive understanding of the model’s ability to predict student outcomes accurately. Integrating these supplementary criteria with established evaluation methodologies renders the model’s long-term projections more reliable and valid.

### Summary of method contributions

To solve the challenges associated with forecasting students’ performance, the GNN-Transformer-InceptionNet (GNN-TINet) model introduces the following additional features:

1. First, a unified architecture that captures multi-scale features, temporal patterns, and other sophisticated data interactions using GNN, Transformer, and InceptionNet layers.
2. Improves data quality in advanced preprocessing using contextual frequency encoding and adaptive imputation.
3. To address the class imbalance and choose relevant characteristics, it employs Hierarchical Contextual Feature Scoring (HCFS) and Cluster-Based Class Expansion (CBCE).
4. Novel Evaluation Criteria: Proposes PCS and LIF to Assess Models’ Long-Term Effectiveness.

With all these factors included, the model can better forecast how well children would do in different types of classrooms.

## Simulation results and discussion

A machine with 32 GB of RAM and an Intel Core i7 9th Generation quad-core CPU was used to conduct extensive simulations. This system can handle deep learning techniques and big datasets with enough processing power. The proposed method employed a learning rate of 0.001, 64 batches, and 100 training epochs to get better results. The training was stopped after five epochs if the validation loss did not improve, and the dropout rate was set at 0.5 to prevent overfitting. While maintaining computational efficiency, these adjustments improved the model’s ability to predict student success.

The data undergoes basic preprocessing to address missing values, convert categorical variables, normalize numerical characteristics, and detect outliers. Following this preparation, dataset analysis informs the subsequent operations. After the dataset analysis, exploratory data analysis is conducted.

**Fig 2:**
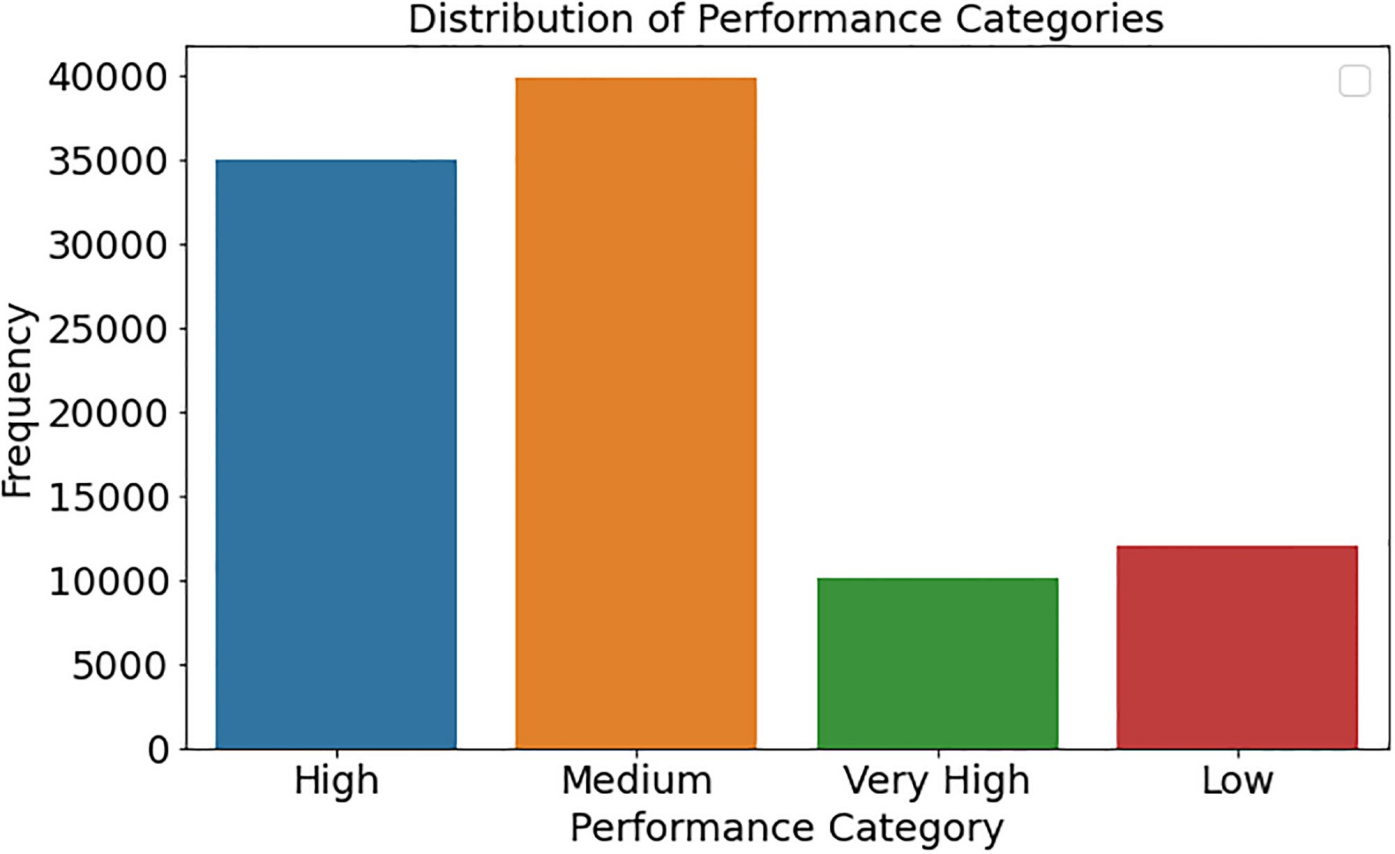
Distribution of performance categories among students.

Fig 2 illustrates the dataset’s distribution of performance categories across students. The x-axis represents the performance categories, while the y-axis denotes the frequency of pupils inside each category. This illustration discerns the number of students categorized inside each performance level, including Very High, High, Medium, and Low. By analyzing bar heights, readers may evaluate the equilibrium of student performance and pinpoint areas for pedagogical improvement. The accurate labelling of the axes and general structure enhances the chart’s readability and elucidates the dataset’s performance landscape.

**Fig 3:**
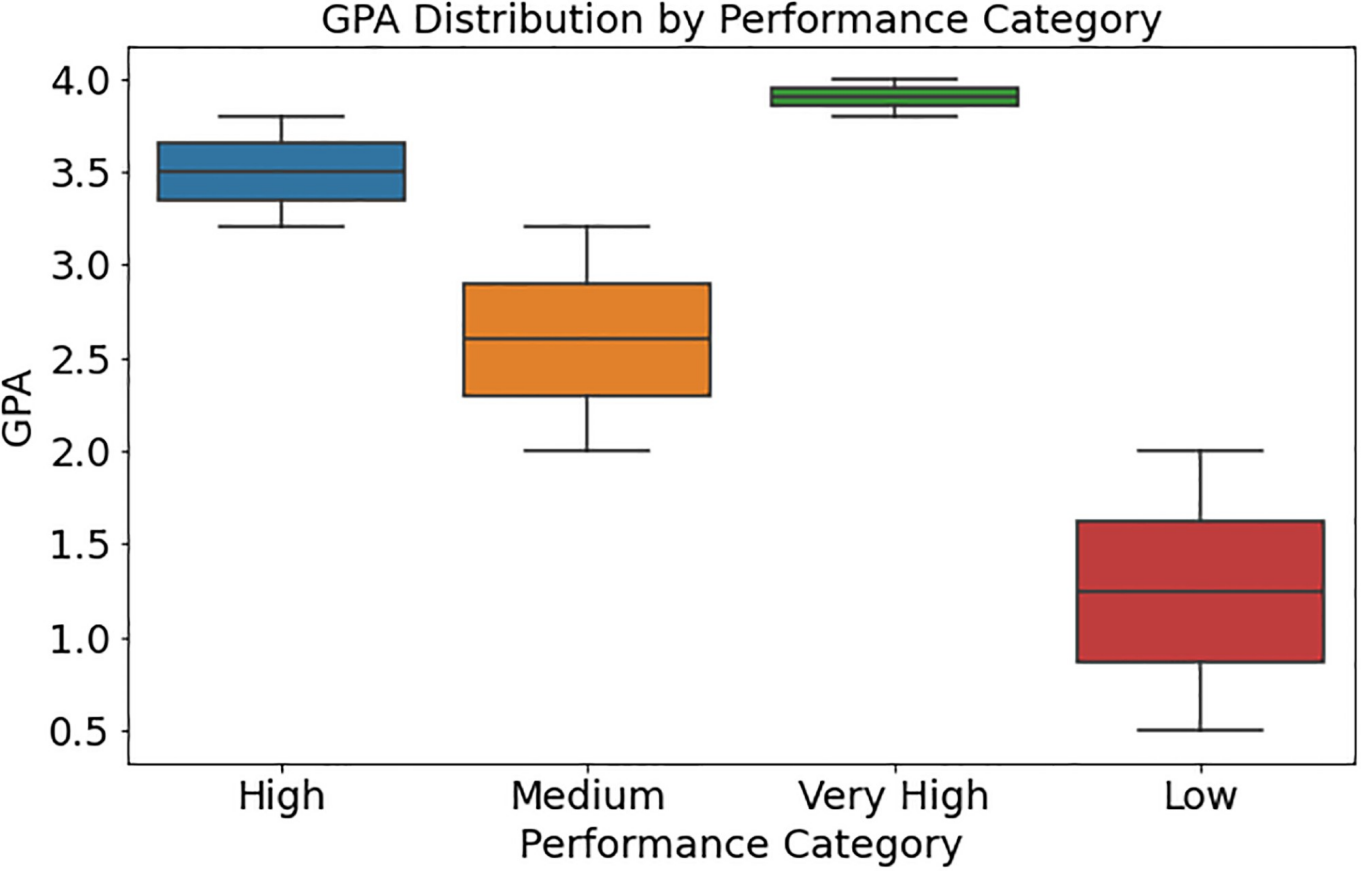
Distribution of student performance categories based on GPA.

Fig 3 demonstrates how student performance differs by performance category and GPA distribution. The box plot shows the median GPA, IQR, and outliers for each performance category, which may include “Very High,” “High,” “Medium,” and “Low.” The median line in the box shows the central tendency of GPA within each category. In contrast, the box shows the distribution of GPA values, showing the middle 50% of scores. Outliers—students with substantially different GPAs from the trend in their categories—may be found outside the whiskers. This chart shows students’ academic achievement by overall performance level and how GPA may relate to different categories. This data may help identify student strengths and weaknesses and provide educational initiatives and assistance.

**Fig 4:**
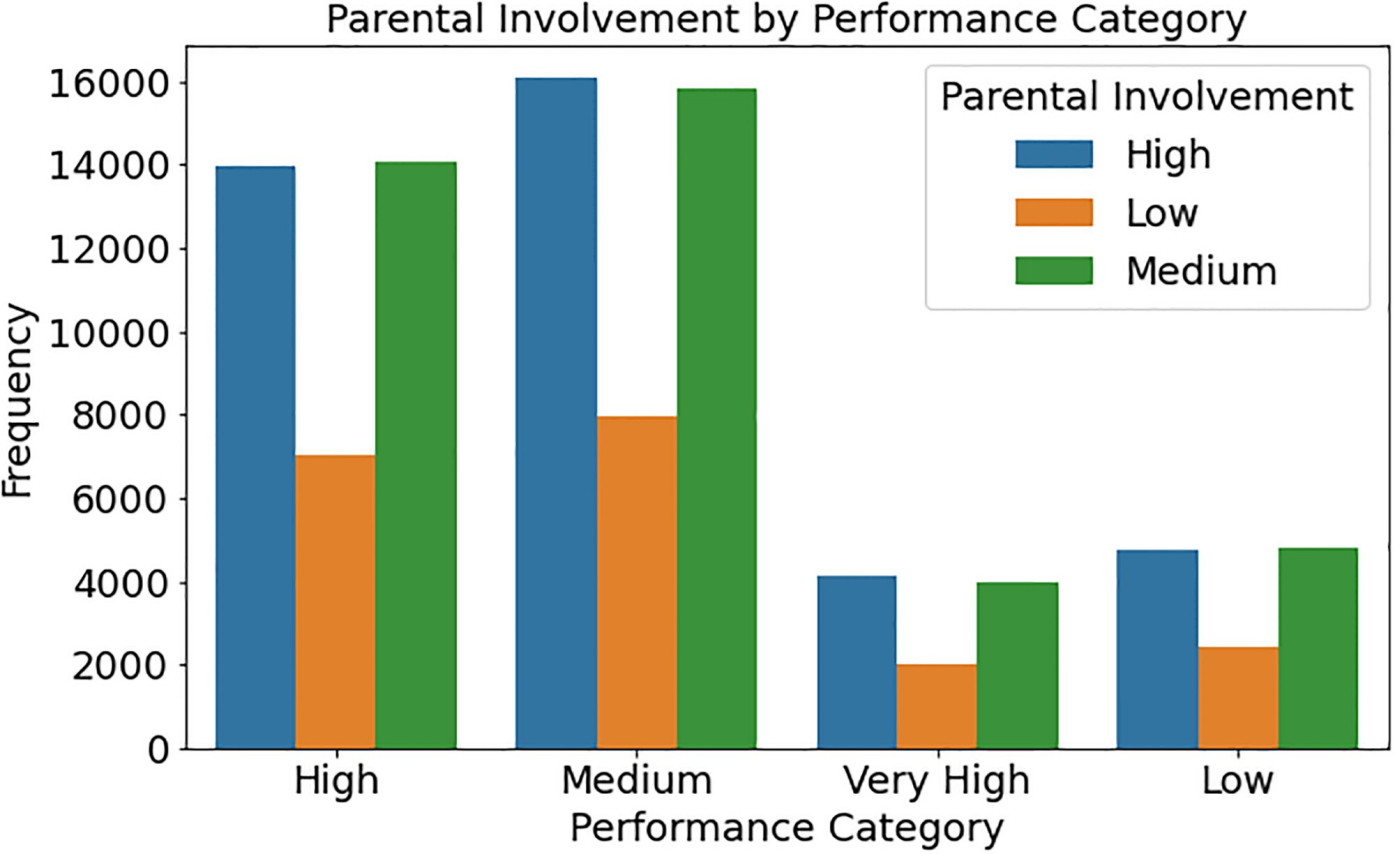
Distribution of student performance categories based on parental involvement levels.

Parental engagement distribution of student achievement categories is shown in Fig 4. The counterplot shows the frequency of pupils in each performance category, from “Very High” to “Low,” with distinct colours denoting parental participation (“High,” “Medium,” and “Low”). The figure shows how parental participation affects student achievement. If the plot shows a greater frequency of “Very High” performers with “High” parental participation, it demonstrates that parental engagement may improve academic achievement. If “Low” performers have “Low” parental participation, it may imply that a lack of parental support may lead to low performance.

**Fig 5:**
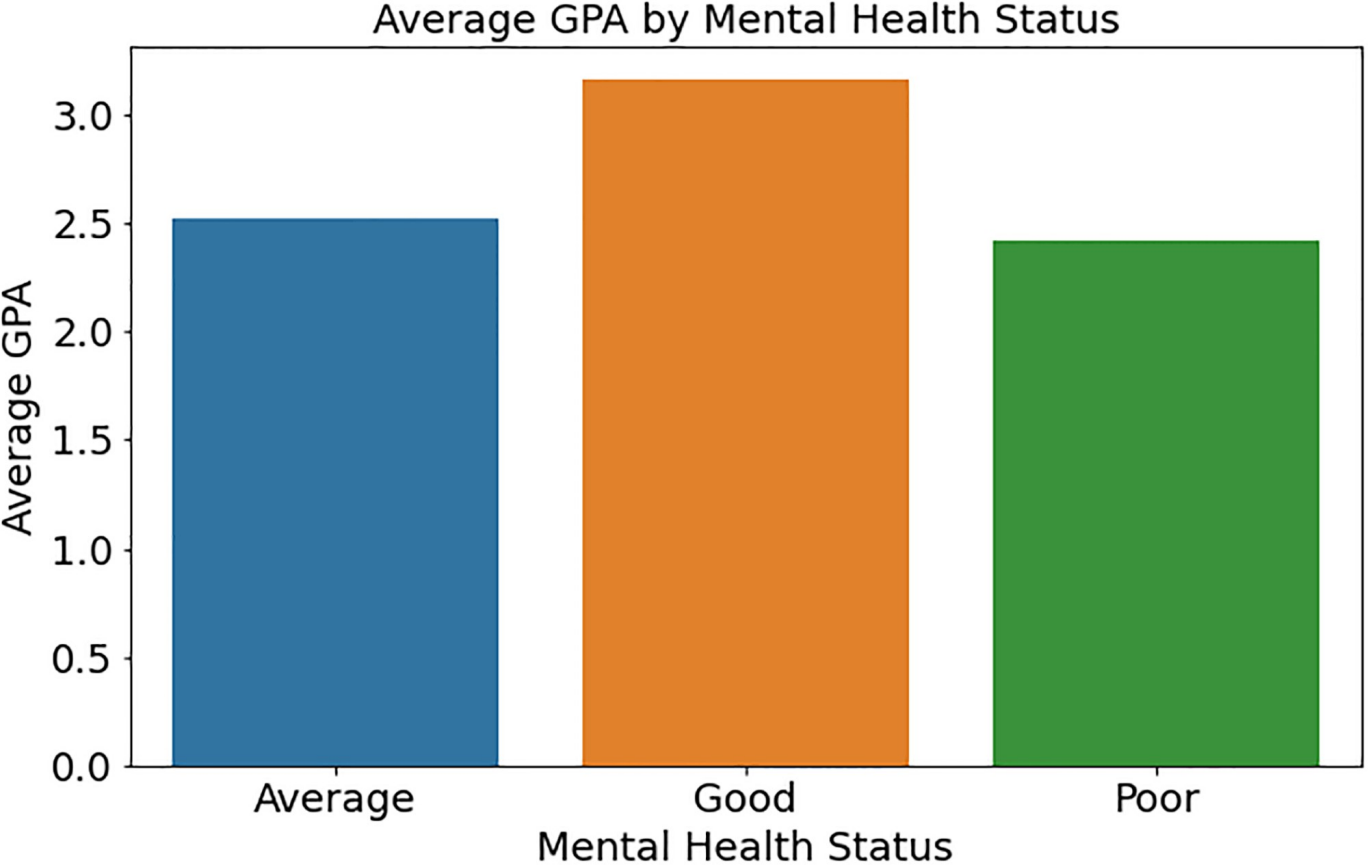
Average GPA by mental health status.

Fig 5 shows students’ average GPA by mental health condition. The bar plot shows how mental health conditions like “Good,” “Average,” and “Poor” affect students’ average GPA. Each bar shows the mean GPA for students in each mental health condition group, comparing performance. This statistic suggests that students with “Good” mental health have higher average GPAs than those with “Average” or “Poor” mental health. This link may reveal the relevance of mental well-being in education and its impact on student performance.

**Fig 6:**
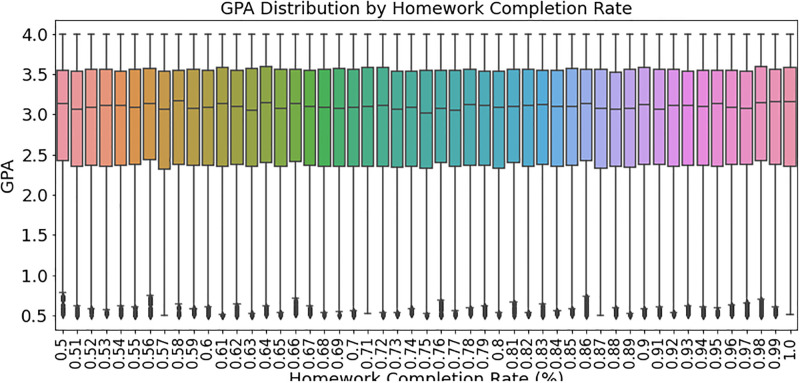
Boxplot of GPA by homework completion rate, showing that higher completion rates correlate with higher GPAs. Outliers indicate variability in performance.

Fig 6 shows student GPA vs homework completion rate. The boxplot shows how homework completion affects GPA. The line within each box shows the median GPA for a specific range of homework completion rates. The whiskers reflect the remaining data range, whereas points outside them may be outliers. The statistics show that students who regularly do their assignments have higher GPAs. Despite high or low assignment completion rates, outliers may have GPAs that are considerably different from their contemporaries. This chart shows how homework completion affects student performance, helping educators and policymakers improve academic success.

**Fig 7:**
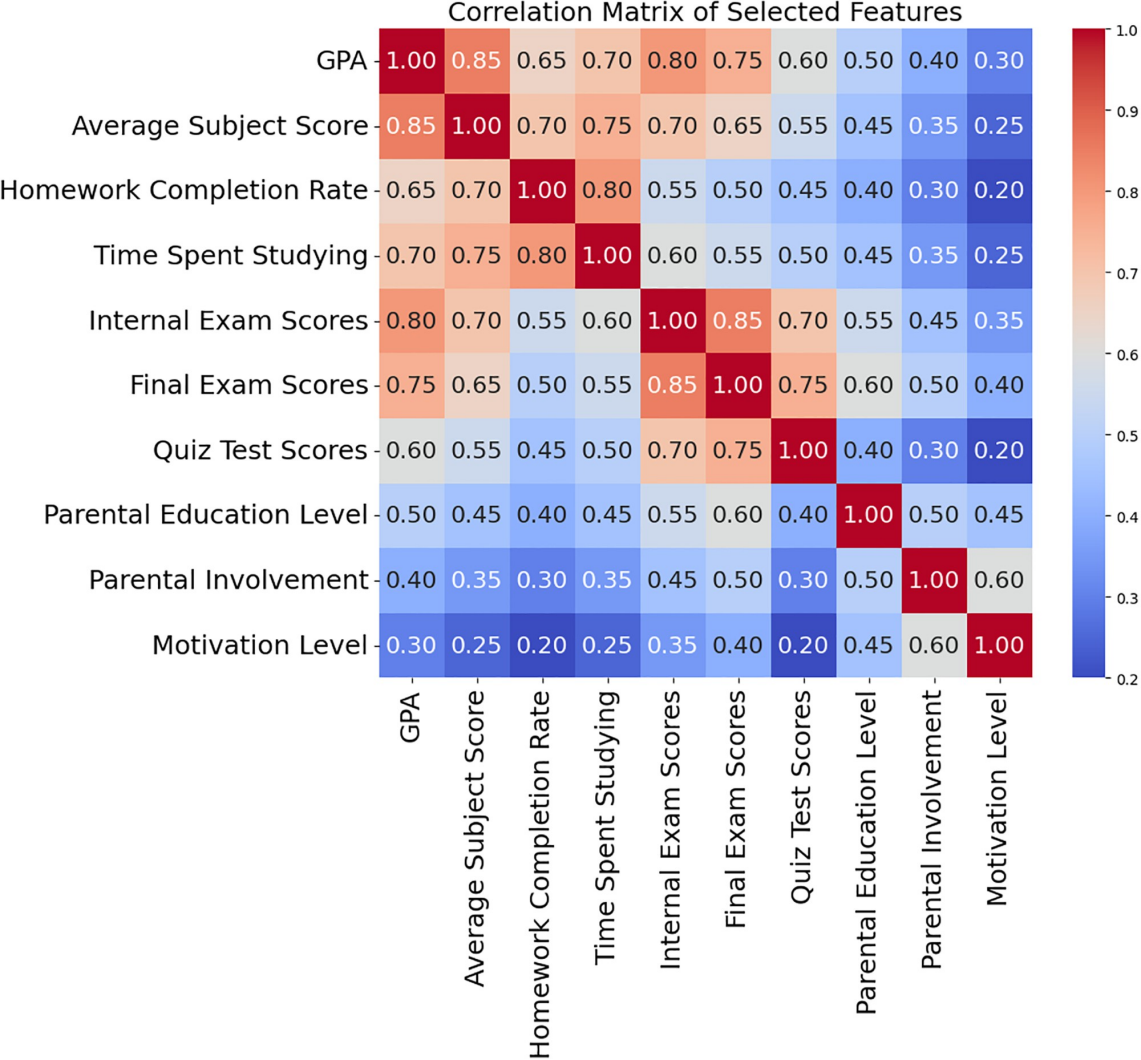
Correlation matrix of selected features.

Fig 7 shows the correlation matrix for early student performance prediction characteristics. Each heatmap column indicates the correlation coefficient between two attributes, ranging from -1 to 1.1 represents a complete positive correlation, suggesting that when one characteristic rises, the other does, too; -1 indicates a perfect negative correlation, where one trait increases and the other decreases. This matrix shows positive connections between GPA, Final Exam Scores, and Internal Exam Scores, implying that students who do well on examinations have better GPAs. Time spent studying also correlates with assignment completion rate, showing that students who study are more likely to finish their homework regularly. However, parental education and motivation levels have modest correlations with academic achievement measures, indicating that they may affect student outcomes but are less directly linked than more linked factors. This correlation matrix helps analyze and choose features for predictive modelling by showing how different variables affect student performance.

**Fig 8:**
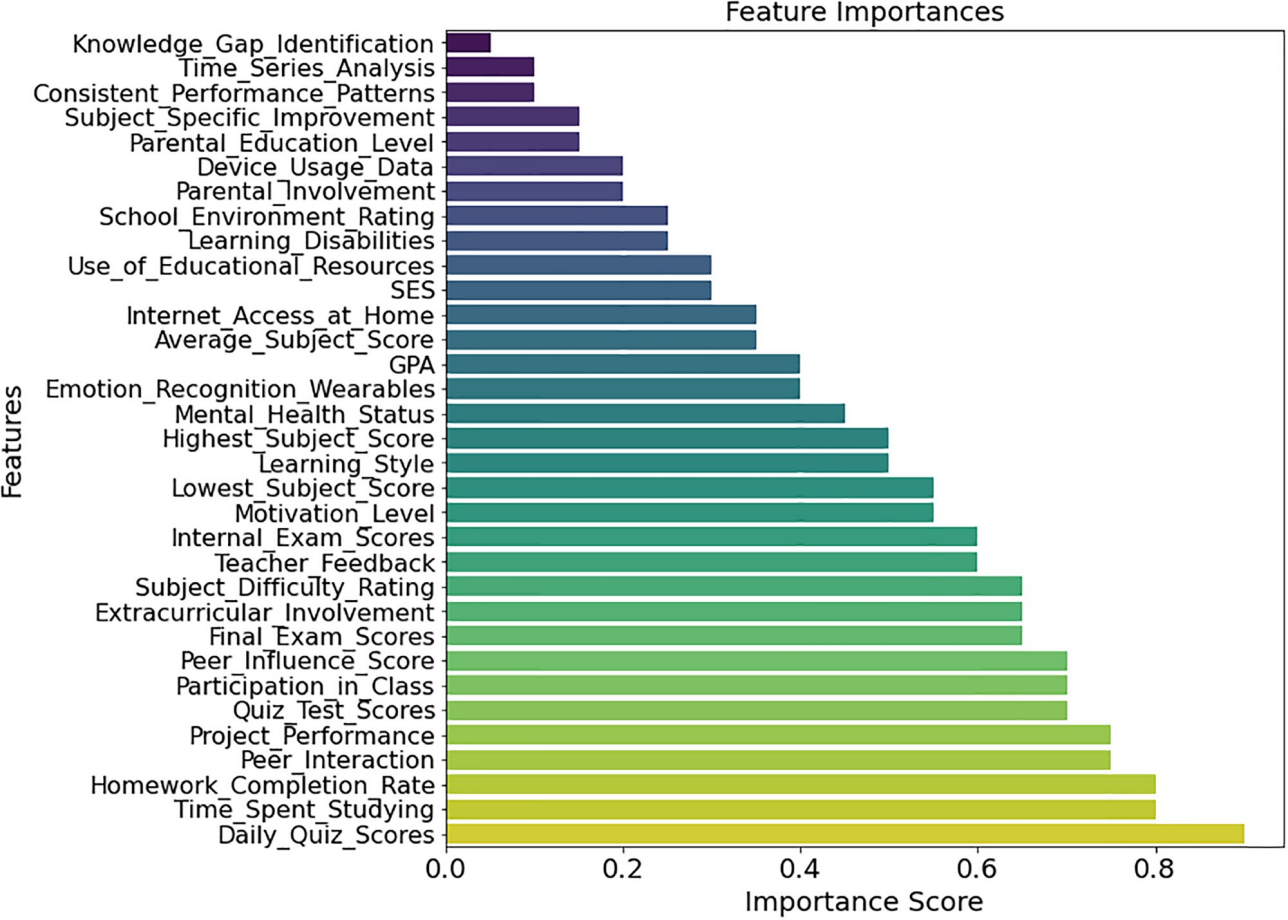
Feature importance’s based on HCFS.

Fig 8 shows how Hierarchical Contextual Feature Scoring (HCFS) measures feature significance in predicting early student success. The bar plot shows feature significance ratings in increasing order. The figure shows that “Peer Influence Score,” “Learning Disabilities,” and “Parental Education Level” have lower significance ratings, indicating they may predict student performance less. In contrast, “GPA,” “Average Subject Score,” and “Time Spent Studying” have greater significance values, reflecting their relevance in student outcomes. This graphic shows which prediction model characteristics are more important and the underlying variables that affect student success. Understanding the proportional significance of these qualities helps educators and policymakers choose interventions and support systems to improve student outcomes.

Table 3 compares the proposed GNN-TINet approach against GritNet, SVM, DenseNet, ResNet, and CNN. The table shows each model’s early student performance prediction accuracy, precision, recall, F1 score, log loss, area under the curve (AUC), Matthews correlation coefficient (MCC), specificity, balanced accuracy, predictive consistency score, learning impact factor, and Hamming loss. From the table, GNN-TINet’s 98.5% accuracy is more significant than all other approaches, demonstrating its better predictive power. GNN-TINet’s accuracy and recall scores indicate that it generates accurate predictions and detects relevant occurrences, decreasing false negatives. Log loss and AUC measures show the model’s dependability, with GNN-TINet having lower log loss and greater AUC than other approaches.

**Table 3:** Performance evaluation of proposed method GNN-TINet and existing methods.

Performance Metric	GNN-TINet	GritNet [20]	SVM [23]	DenseNet [5]	ResNet [28]	Enhanced CNN-1D [41]	Decision Tree [40]	MLP [42]	XG-Boost Hybrid [43]
Accuracy (%)	98.5	90.0	85.5	89.0	88.0	89.0	90.0	91.0	92.0
Precision (%)	97.5	88.0	84.0	87.5	89.0	86.5	87.0	88.0	89.0
Recall (%)	98.0	89.0	86.0	88.0	90.5	87.0	87.5	88.5	90.0
F1 Score	97.8	88.5	85.0	87.7	89.5	86.8	87.3	88.2	89.4
Log Loss	0.05	0.12	0.15	0.11	0.11	0.13	0.14	0.12	0.10
AUC	0.99	0.85	0.82	0.83	0.84	0.82	0.83	0.85	0.88
MCC	0.95	0.75	0.70	0.76	0.77	0.74	0.73	0.75	0.78
Specificity (%)	97.0	85.0	80.0	82.5	83.0	81.0	82.0	83.5	84.5
Balanced Accuracy (%)	97.5	87.0	83.0	85.5	85.5	84.0	85.0	86.0	87.0
PCS	0.92	0.80	0.78	0.79	0.78	0.77	0.78	0.79	0.81
LIF	0.90	0.76	0.72	0.73	0.74	0.73	0.74	0.75	0.76
Hamming Loss	0.02	0.10	0.12	0.09	0.11	0.10	0.11	0.10	0.09

**Fig 9:**
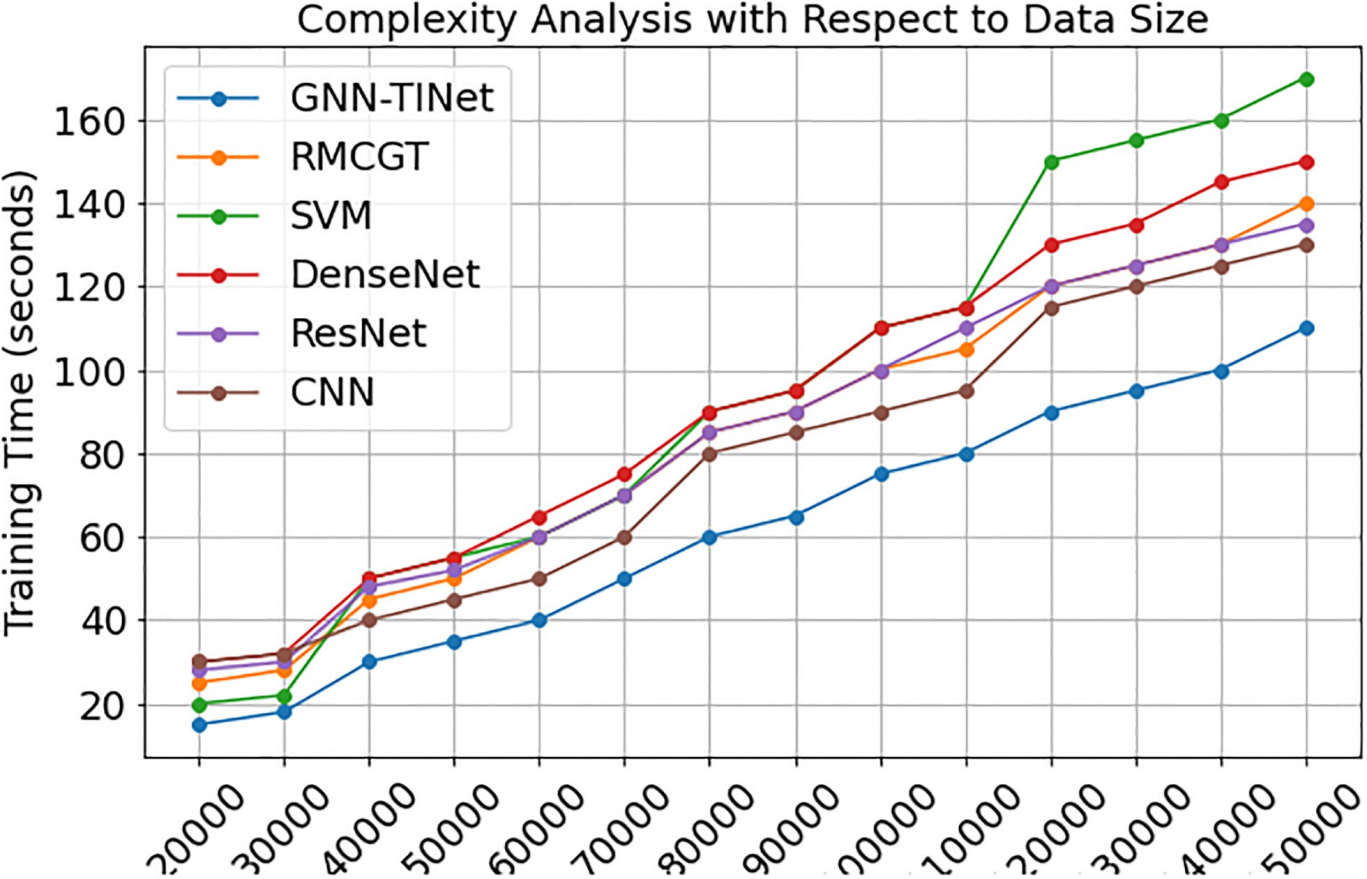
Training and testing accuracy and loss of the GNN-TINet model over 30 epochs.

The GNN-TINet model’s 30-epoch training and testing accuracy and loss are displayed in Fig 9. The left subplot represents training/testing model accuracy. The training accuracy slowly improves to 98.8%, indicating model learning from training data. The model generalizes well to additional data as testing accuracy rises to 98.8% in the final epoch. The right subplot indicates training/testing loss. The model becomes more accurate when the training loss decreases from 0.72 to 0.10. The testing loss drops to 0.09, confirming that the model matches the training data and performs well on the test set. The picture shows that the GNN-TINet model predicts early student performance with high accuracy and low loss values for training and testing. Real-world scenarios suit the model.

Table 4 displays the GNN-TINet model’s parameter sensitivity analysis, which shows how hyperparameters affect performance measurements. Learning rate, batch size, dropout rate, and training epochs are all examined with different values. The data indicates that the GNN-TINet model’s accuracy reaches 98.5% with specified parameters, highlighting the need for hyperparameter adjustment for best performance. A learning rate of 0.001 and a batch size of 64 results in the highest accuracy and F1 score, making them suitable for model training. The picture also depicts how dropout rate and epochs influence model performance, emphasizing that tuning may significantly impact log loss and area under the curve. It concludes that carefully evaluating these hyperparameters enhances the GNN-TINet model’s anticipated early student performance, giving valuable insights for practitioners looking to replicate or improve these results.

**Table 4:** Parameter sensitivity analysis for GNN-TINet model.

Parameter	Value	Accuracy (%)	F1 Score	Log Loss	AUC
Learning Rate	0.001	98.5	97.8	0.05	0.99
	0.0005	97.8	96.5	0.07	0.95
	0.01	96.2	95.0	0.10	0.90
Batch Size	32	97.5	96.2	0.06	0.96
	64	98.5	97.8	0.05	0.99
	128	97.0	95.5	0.08	0.94
Dropout Rate	0.2	98.0	97.0	0.06	0.96
	0.5	98.5	97.8	0.05	0.99
	0.7	97.2	96.0	0.08	0.93
Epochs	50	97.5	96.5	0.06	0.95
	100	98.5	97.8	0.05	0.99
	150	97.0	95.5	0.07	0.92

**Fig 10:**
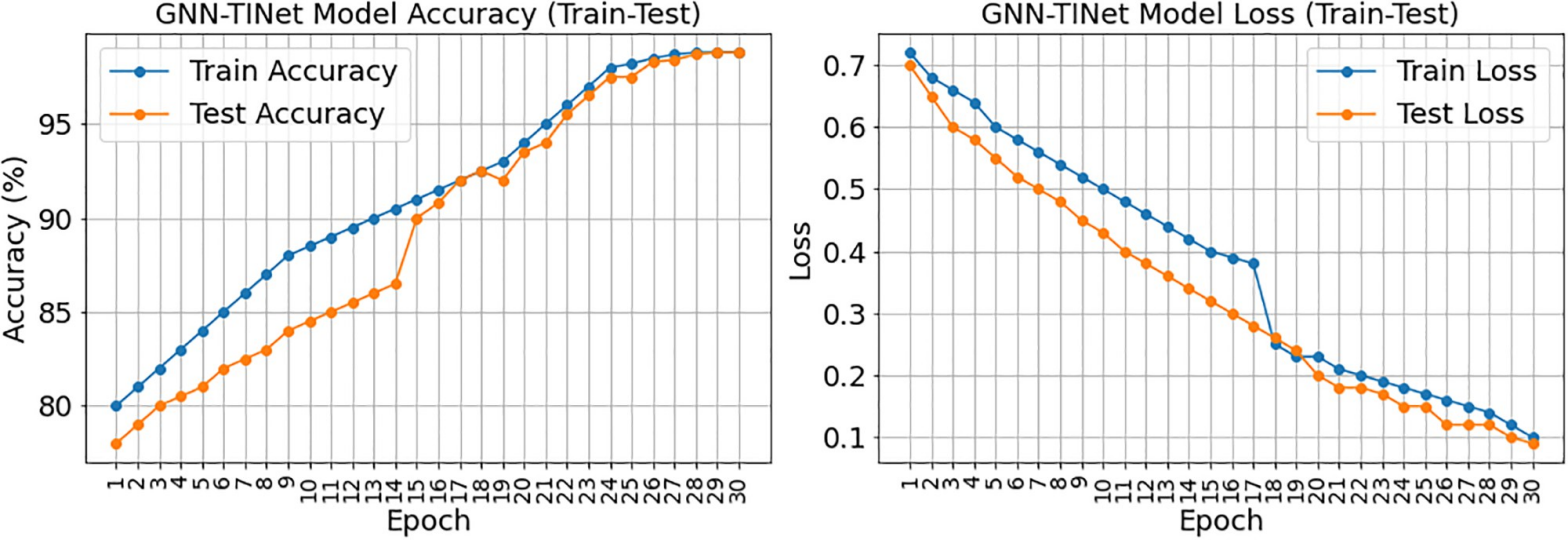
Complexity analysis of the proposed and existing method.

Fig 10 shows the complexity analysis of several machine learning approaches with increasing data quantities. The graph compares GNN-TINet, GritNet, SVM, DenseNet, ResNet, and CNN training periods (in seconds) from 20,000 to 150,000 cases. According to the chart, GNN-TINet has the lowest training times across all data sizes. This implies that it can manage additional datasets with greater efficacy and scalability. Conversely, the training time for SVM and DenseNet increases significantly as the size of the dataset increases, particularly for larger datasets. This visualization emphasizes the most efficient method for large-scale data processing: GNN-TINet. Constraints revealed by trends can expose other methods’ computational difficulty and practical consequences. This number emphasizes the need to choose effective methods to project large amounts of data.

## Conclusion and future work

This study improves educational data mining by developing an effective strategy for forecasting students’ early performance. The GNN-Transformer-InceptionNet (GNN-TINet) model utilizes GNNs, transformers, and the Inception architecture to interpret intricate data on student performance. Thanks to these intricate models, the GNN-TINet model can process-relational and contextual data, which improves its ability to generalize to different learning contexts and make accurate predictions. Following a battery of simulations, the GNN-TINet model outperformed conventional machine learning models in terms of accuracy (98.5%), recall (99%) and F1 score (98). Combining contextual awareness and multi-scale feature engineering enables the model to comprehend intricate patterns and relationships within educational data. By analyzing demographics, behavioural traits, academic performance, and other multi-dimensional attributes, the GNN-TINet model aids educators in foreseeing and enhancing student results. Beyond the accuracy of forecasts, the method gives teachers and legislators helpful information about students’ development. The GNN-TINet model accurately predicts performance patterns, allowing personalized interventions that provide responsive and helpful learning environments. These treatments promote a holistic view of student development and achievement by addressing emotional, cognitive, and social barriers to achievement.

There are downsides to this study despite the positive results. Statistics derived from public sources may not fairly portray the unique features of different classroom settings. The results obtained using the model in contexts different from the one used for this study might differ. Improving models for behavioural analysis and dropout prediction, expanding data sources, or real-time learning analytics could result from removing these limitations.
